# Supplementary material for: Shavenbaby and Yorkie mediate Hippo signaling to protect adult stem cells from apoptosis
Source: Nat Commun. 2018 Nov 30;9:5123. doi: 10.1038/s41467-018-07569-0 (PMC6269459; doi:10.1038/s41467-018-07569-0)
Supplement: Supplementary file 1 — Supplementary Information [file 41467_2018_7569_MOESM1_ESM.pdf]

Supplementary Information

Shavenbaby and Yorkie mediate Hippo signaling to protect adult stem cells from apoptosis

Jérôme Bohère[1], Alexandra Mancheno-Ferris[1], Sandy Al Hayek[1,4,5], Jennifer Zanet[1], Philippe Valenti[1], Kohsuke Akino[2], Yuya Yamabe[2], Sachi Inagaki[3], Hélène Chanut-Delalande[1], Serge Plaza[1], Yuji Kageyama[2,3], Dani Osman[4,5], Cédric Polesello[1*] & François Payre[1]*

* Correspondence and requests for materials should be addressed to C.P. or F.P. (email: cedric.polesello@univ-tlse3.fr, francois.payre@univ-tlse3.fr)

This file includes:

Figs. S1 to S9

Supplementary Methods

Supplementary References

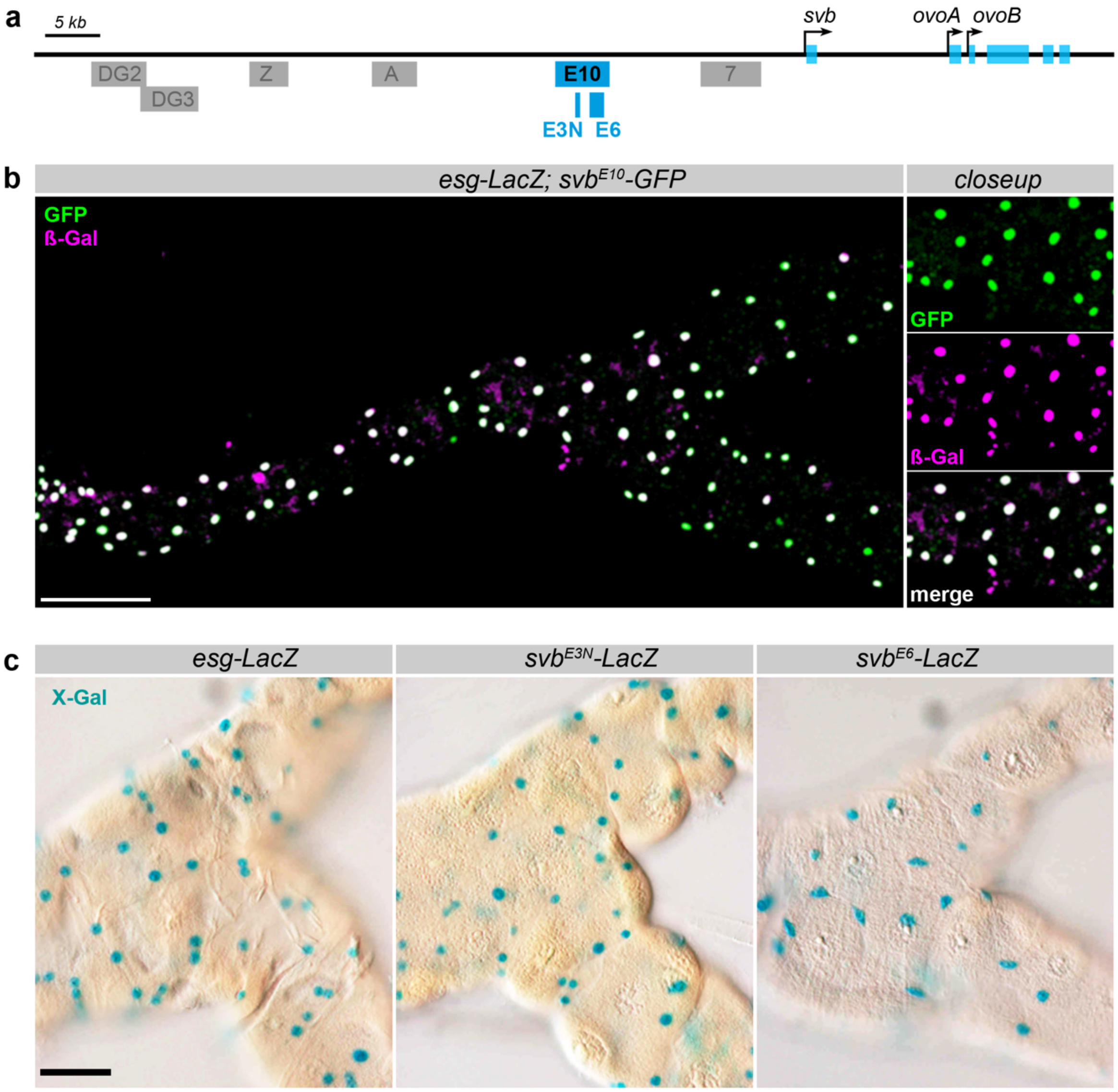

Supplementary Figure 1: *svb* enhancers driving expression in RNSCs

(a) Schematic representation of the *svb* locus, with validated enhancers indicated as boxes; those driving expression in RNSCs are in blue. **(b)** The *svb*^{E10} enhancer (*svb*^{E10}*-GFP*) is active in RNSCs, as shown by co-expression with *esg-LacZ* (red). **(c)** The shorter elements *svb*^{E3N} and *svb*^{E6} are each sufficient to drive expression in RNSCs as revealed by X-Gal staining and comparison with *esg-lacZ*. Scale bar is 50 µm.

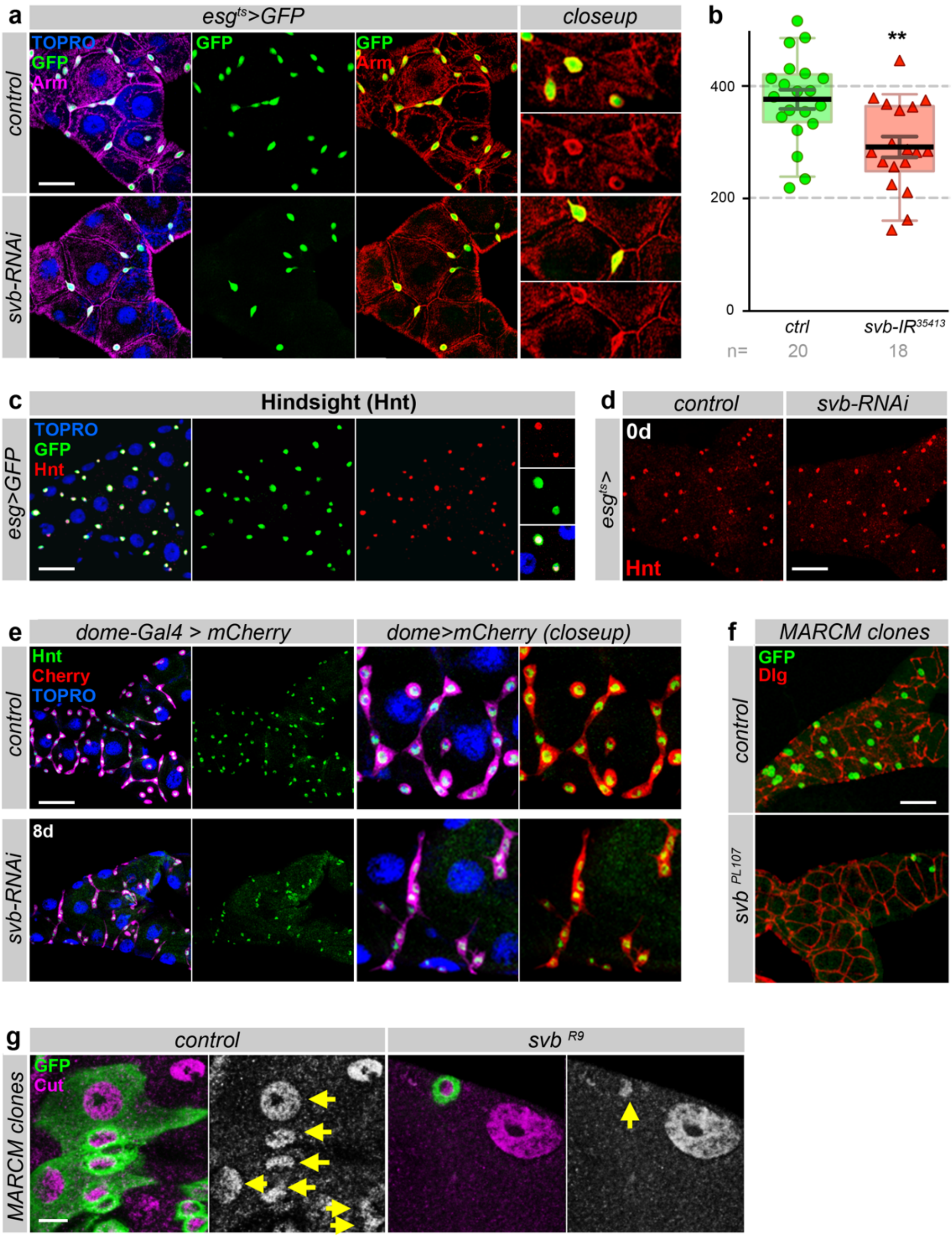

Supplementary Figure 2: *svb* is required for RNSC maintenance

(a) *esg*^{ts}**-**driven expression of *svb*-RNAi in the adult (8 days of treatment) depletes the renal stem cell compartment, as shown by staining against GFP (green), Armadillo (Arm, purple or red) and DNA (blue). **(b)** effect of a second RNAi targeting non-overlapping regions of the *svb* mRNA on RNSCs (8 days, p=0.0042). **(c)** The transcription factor Hindsight (Hnt, red) is expressed in *esg*-positive RNSCs (green). **(d)** Staining for Hnt (red) 3 days after hatching (referred to as day 0 in Fig. 1) confirms the presence of adult RNSCs before inducing *esg*^{ts} *svb-RNAi* expression. **(e)** *dome-MESO-Gal4* drives specific expression (mCherry, red) in RNSCs, as shown by colocalization with Hnt (green). *dome-MESO*-driven expression of *svb-RNAi* leads to a reduction in the number of Hnt-positive RNSCs. Nuclei are in blue. **(f)** Mosaic clones (MARCM) of control and *svb*^{PL107} mutant cells, labelled with GFP (green), 25 days after clone induction. The cell contour is revealed by staining against Dlg (red). **(g)** closeup of MARCM clones, labelled by GFP (green) and stained for Cut (purple). In both control and *svb*^{R9} mutant, clonal cells with a small diploid nucleus display moderate levels of Cut, a characteristic feature of RNSCs [44]. Values are presented as average +/- standard error of the mean (SEM) and boxes with whiskers (10-90 percentile). P-values are from a Mann–Whitney test (**, P<0,01). Scale bar is 30 µm in all panels, except **g** (3 µm).

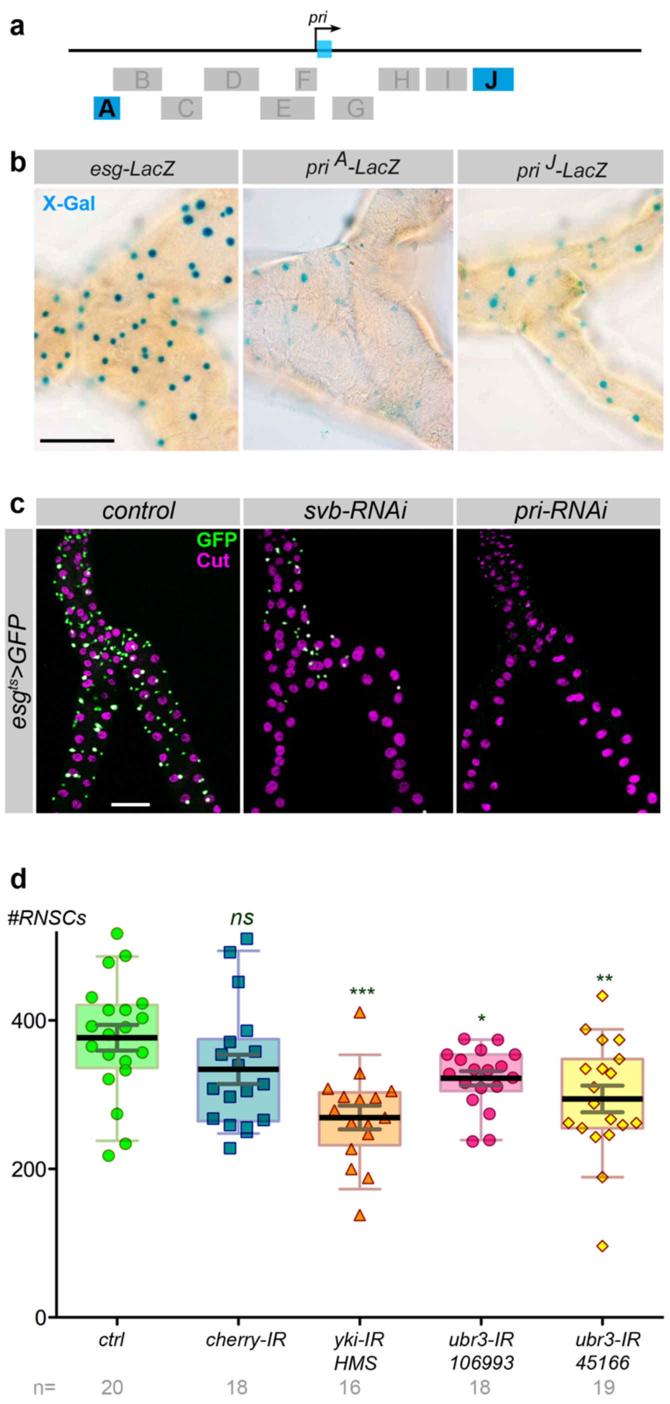

Supplementary Figure 3: Pri expression and function in RNSCs

(a) Drawing of the *pri* locus, with the *priA* and *priJ* enhancers (blue) that are both active in adult RNSCs. **(b)** Fork region of Malpighian tubules of the indicated genotype; X-Gal staining reveals the activity of *priA* and *J* enhancers in subsets of RNSCs in comparison to the *esg-LacZ* reporter. (**c**) esg^{ts}-driven expression of GFP alone (*ctrl*), or together with *svb*-RNAi and *pri*-RNAi. In all pictures GFP is in green and anti-Cut in purple. Scale bar is 30 µm. **(d)** Consequences of esg^{ts}-driven expression of GFP alone (*ctrl*) or together with various RNAi constructs on the number of RNSCs, after 8 days of treatment. Values are presented as average +/- standard error of the mean (SEM) and boxes with whiskers (10-90 percentile). P-values from Mann–Whitney test are *mCherry-RNAi* (0.1134), *yki-RNAi*^{HMS} (<0.0001), *ubr3-RNAi*^{106993} (0,0101), *ubr3-RNAi*^{45166} (0.0022); (ns, P≥ 0.05; *P<0.05, **, P<0,01; ***, P < 0.001). Scale bar is 100 µm.

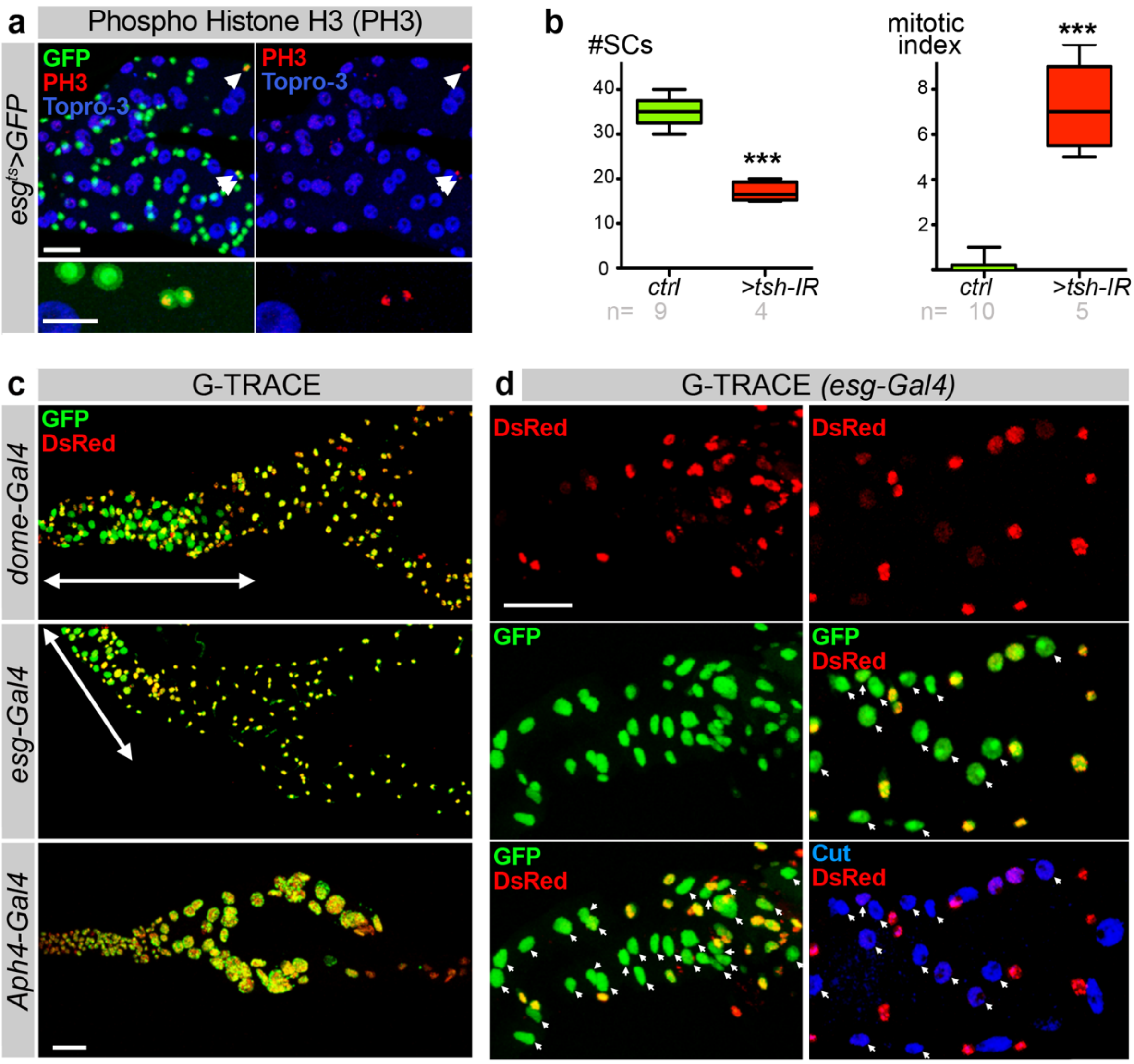

Supplementary Figure 4: Proliferation and lineage of RNSCs

(a) Fork region of the Malpighian tubules, with RNSCs marked by *esg-Gal4>GFP* (green) and mitotic cells by immuno-staining against phospho-Histone H3 (Ser10) (PH3, red). Nuclei were stained by Topro-3 (blue). Scale bar is 30 µm. The bottom panels show a closeup of two RNSCs undergoing mitosis. Scale bar is 10 µm. **(b)** Disrupting renal homeostasis in the adult through expression of *tsh-RNAi* in stellate cells (*c724-Gal4* driver) reduces their number and favour mitotic figures, as followed by PH3 staining. Flies were kept at 18°C before hatching and dissected after 4 days at 29°C. Values are presented as average +/- standard error of the mean (SEM) and boxes with whiskers (10-90 percentile).). P-values from Mann–Whitney test are <0.0001 (***). **(c)** G-TRACE lineage-tracing experiments performed with *esg-Gal4* and *dome-MESO-Gal4,* two drivers specific of RNSCs, or *Aph4-Gal4* that drives specific expression in neighbouring principal cells located in lower tubules. In G-TRACE experiments, the nuclear DsRed protein (Red-Stinger, red) that is expressed under the control of UAS sequences accumulates in cells in which the Gal4 driver is active. The nuclear GFP protein (Stinger, green) -controlled by a flp/FRT cassette- is expressed both in progenitors (Gal4-positive cells) and in their progeny (Gal4-negative cells). Many G-TRACE progeny cells are visible in the lower tubule region (white arrows) for both RNSC drivers, while we never observed progeny from *Aph-Gal4***.** Scale bar is 50 µm **(d)** Closeup of *esg* G-TRACE experiments, focusing on the region of lower tubules. Progeny cells (GFP positive, RFP negative) are highlighted by white arrowheads. On the right panel, tubules were processed for anti-Cut staining (blue), revealing that RNSC progeny differentiate into large polyploid cells principal cells, characterized by high levels of Cut. Flies were raised at 18°C until hatching and kept 21 days at 25°C before dissection. Scale bar is 10 µm.

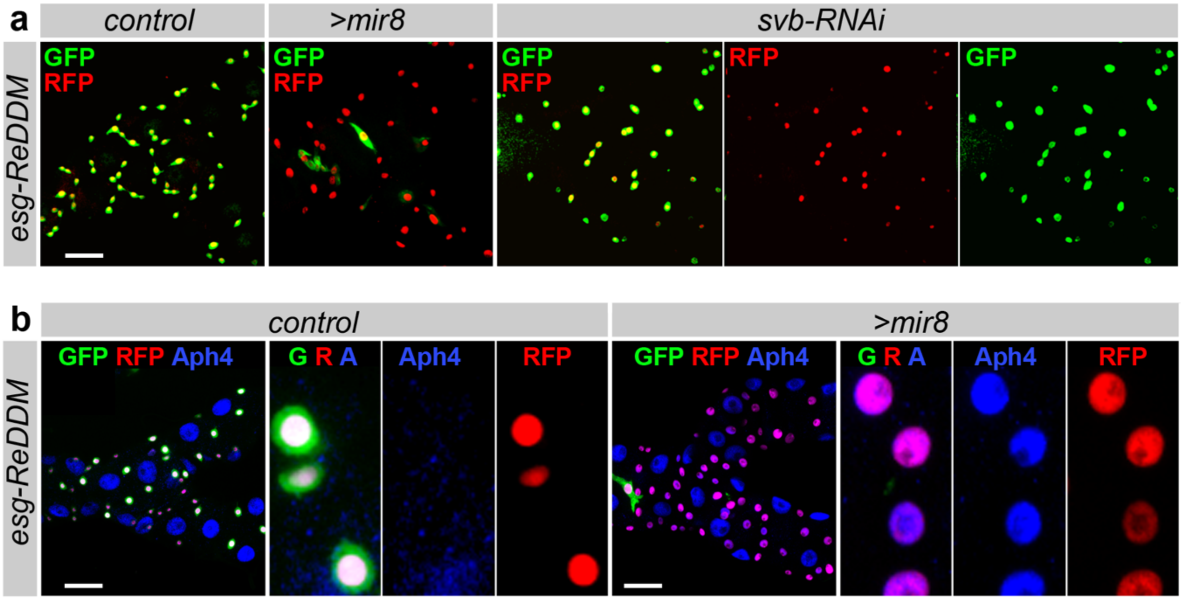

Supplementary Figure 5: consequences of *svb*-RNAi or *mir8* overexpression on RNSCs

(a) *esg-ReDDM* lineage-tracing in control conditions, following *mir8* over-expression or *svb* RNAi-depletion. RNSCs display a green cytoplasm (GFP) and a red nucleus (H2B::RFP), while their progeny only maintain the stable H2B-RFP signal. Pictures are the separate channels of those shown in Fig. 3a. **(b)** *esg-ReDDM* lineage-tracing with or without *mir8* over-expression. Cells prematurely differentiated upon *mir8* overexpression also express the *Aph4-LacZ* reporter (blue), a specific marker of principal cells in lower tubules. Scale bar is 30 µm

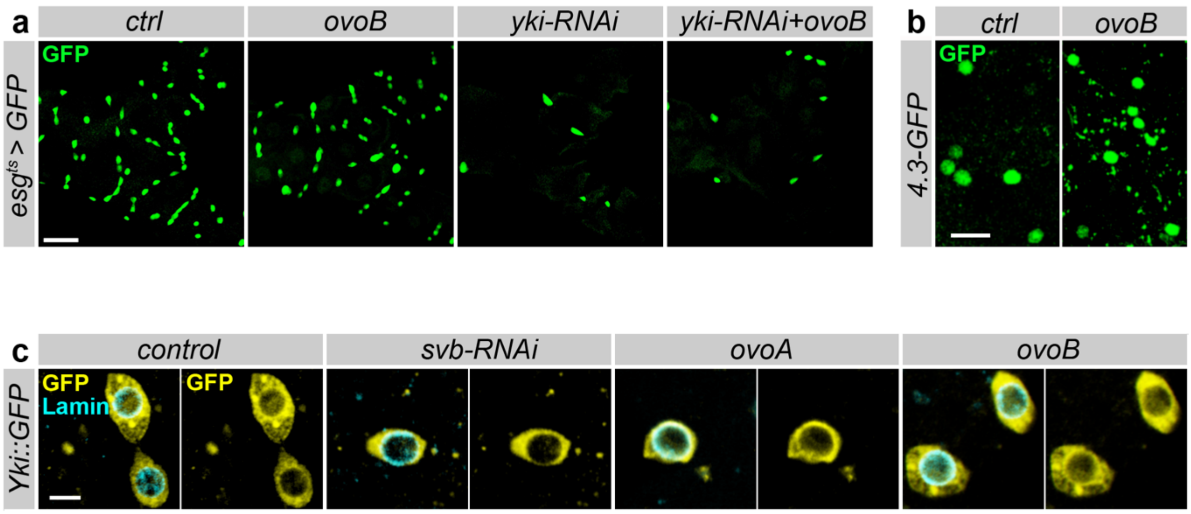

Supplementary Figure 6: effect of Svb on Yki function and subcellular distribution

(a) *esg*^{ts} was used to drive the expression of GFP and the indicated transgenes, for 8 days. Expression of OvoB has not significant effect, in contrast to *yki-RNAi* that induces a drastic drop in RNSC number. This phenotype was not rescued by the co-expression of OvoB. Scale bar is 30 µm. **(b)** *esg*^{ts} was used to drive the expression of *yki*, or *Svb*^{ACT} *(ovoB)* in RNSCs. The expression of *DIAP1* was followed by the activity of the *4.3-GFP* enhancer. Flies were dissected 8 days after induction. Scale bar is 10 µm. **(c)** Svb has not detectable effect on Yki subcellular location. *esg*^{ts} was used to drive the expression of *yki::GFP* in addition to *svb-RNAi*, or *Svb*^{REP} *(ovoA),* or *Svb*^{ACT} *(ovoB)*, in RNSCs. GFP is in yellow and nuclei (Lamin) in cyan. Flies were dissected 8 days after induction. Scale bar is 3 µm

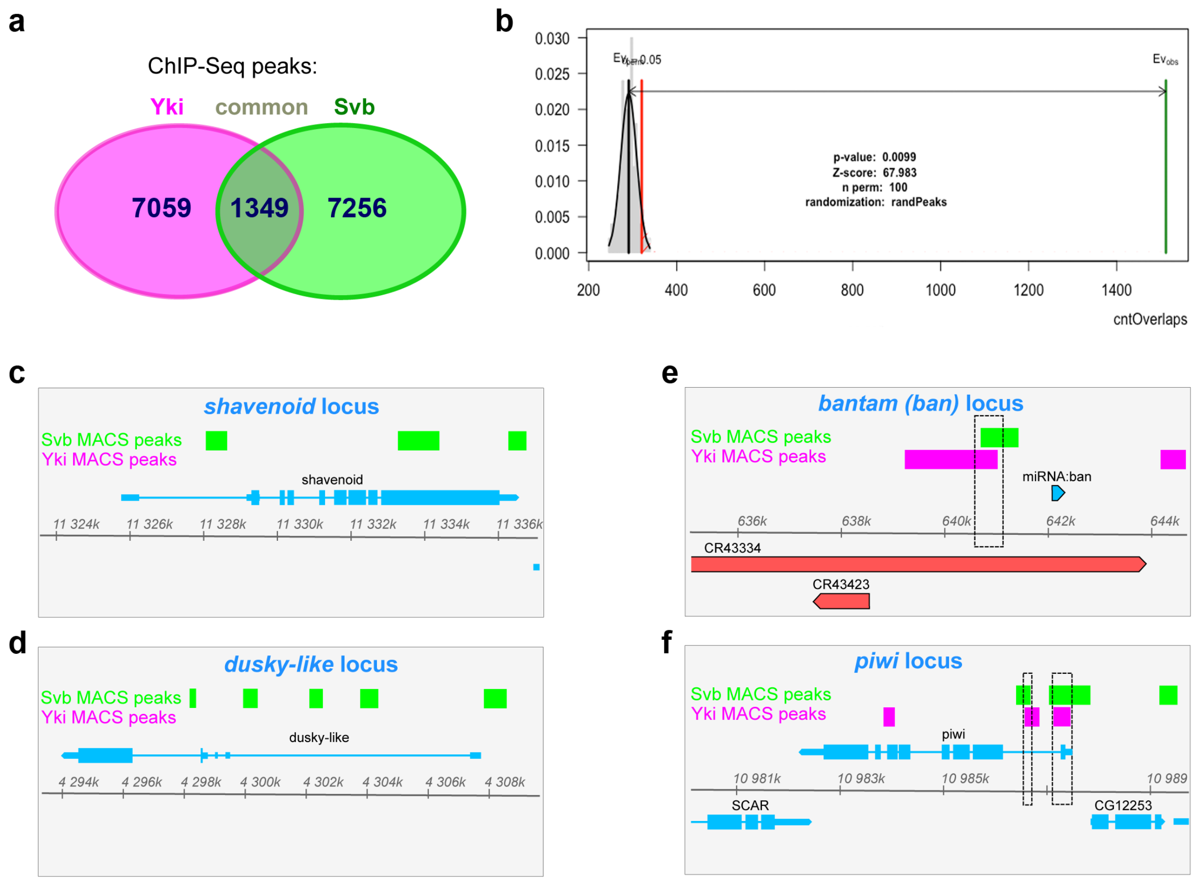

Supplementary Figure 7: comparison of Yki and Svb binding to the *Drosophila* genome

(**a**) Venn diagram representing the genome-wide intersection between Yki and Svb ChIP-seq peaks, in pink and green, respectively. (**b**) Comparison of the observed intersection (Ev_{obs}) between Yki and Svb ChIP-seq peaks *versus* a series of 100 randomly permutated (Ev_{perm}) samples, indicating the significance of the observed intersection (p<0,01). (**c-f**) Location of Svb and Yki binding sites on *shavenoid*, *dusky-like, bantam* and *piwi* loci. MACS peaks of Svb and Yki ChIP-seq are in green and magenta, respectively.

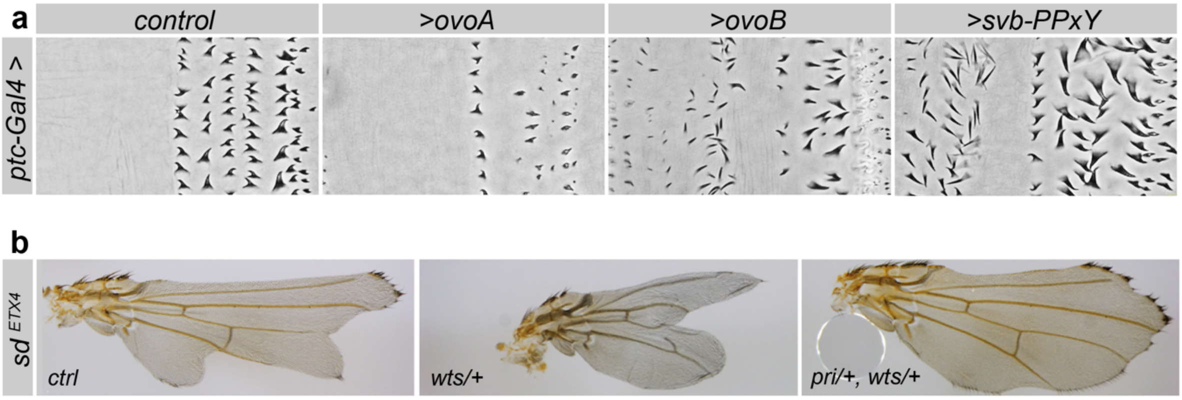

Supplementary Figure 8: Function of Svb/Pri in embryonic and adult tissues
(**a**) cuticles of embryos expressing different forms of the Svb transcription factor under the control of the *ptc-Gal4* driver. **(b)** Adult wings were also used to test genetic interactions between *pri* and *wts* in the context of sd^{ETX4} males.

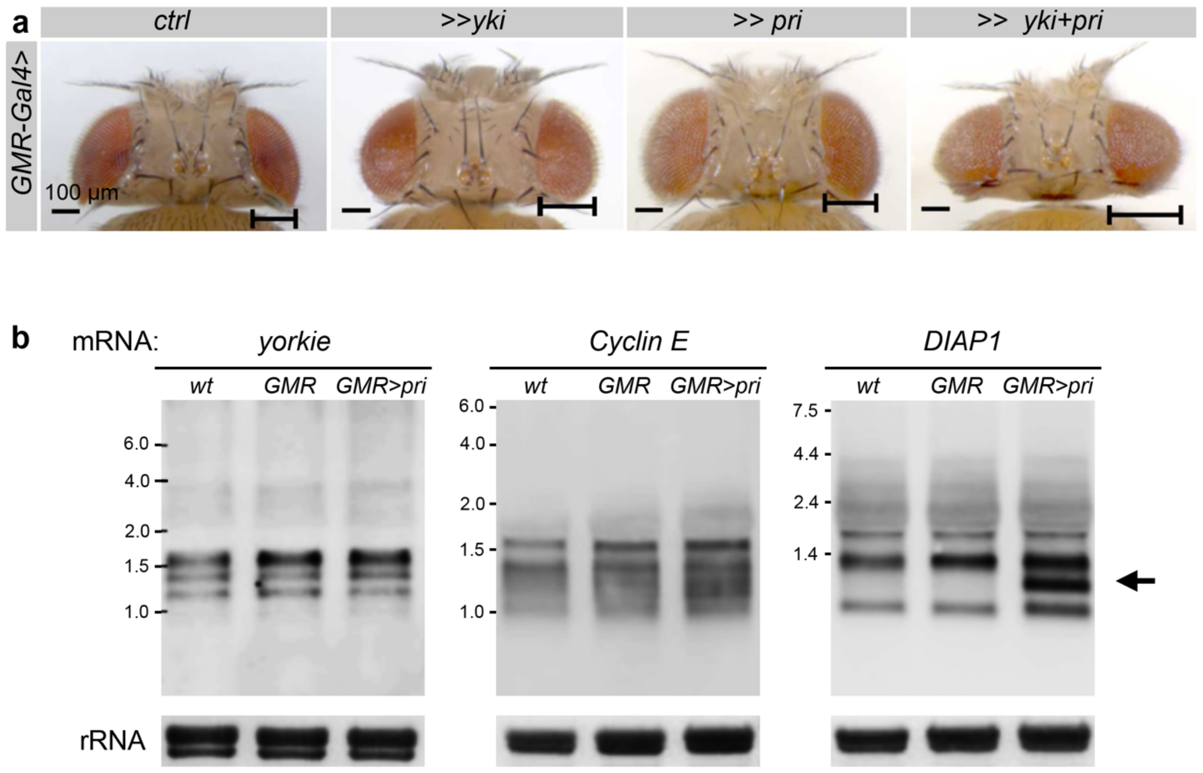

Supplementary Figure 9: *pri* induces overgrowth and activates *DIAP1* expression
(a) Adult head cases dissected form flies expressing the indicated transgenes in the eye, under the control of *GMR-Gal4*. **(b)** Northern blots of adult head mRNAs revealing the expression of *yorkie, Cyclin E* and *DIAP1* in *wild-type (wt), GMR-Gal4 (GMR>)* and *GMR-Gal4, UAS-pri (GMR>pri)* flies. The black arrow highlights a strong upregulation in *DIAP1* transcripts upon *pri* overexpression.

Uncropped scans of Western and Northern blots

Figure 7a

PANEL#1

IP anti GFP

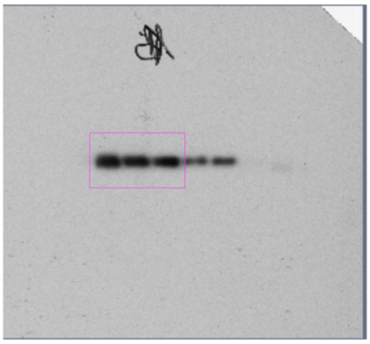

anti-HA

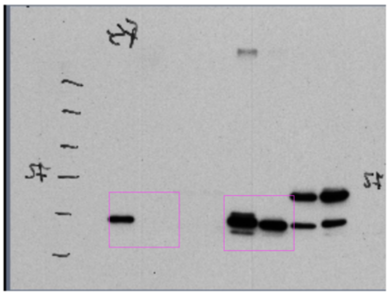

PANEL#2

IP anti GFP

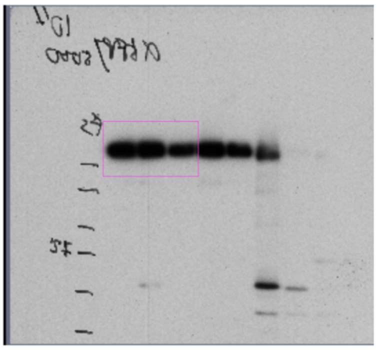

anti-HA

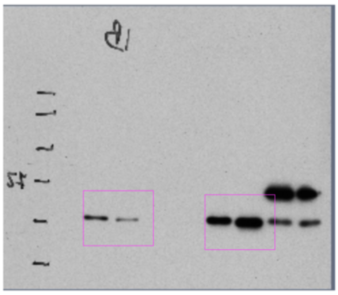

PANEL#3

IP anti GFP

anti-HA

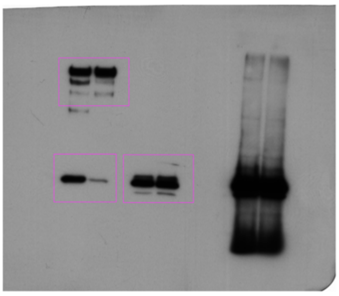

PANEL#4

IP anti anti HA

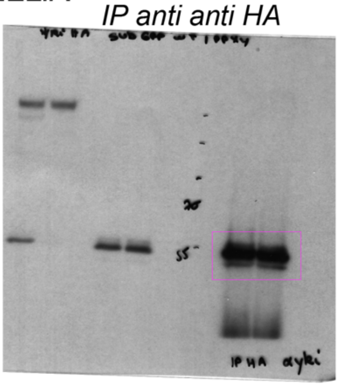

anti-GFP

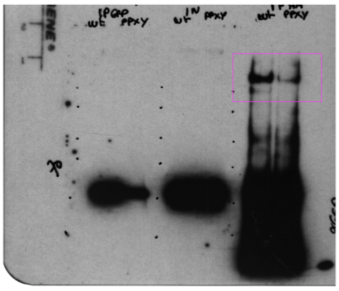

anti-GFP

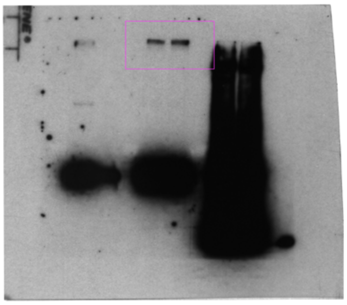

Supplementary Figure 9

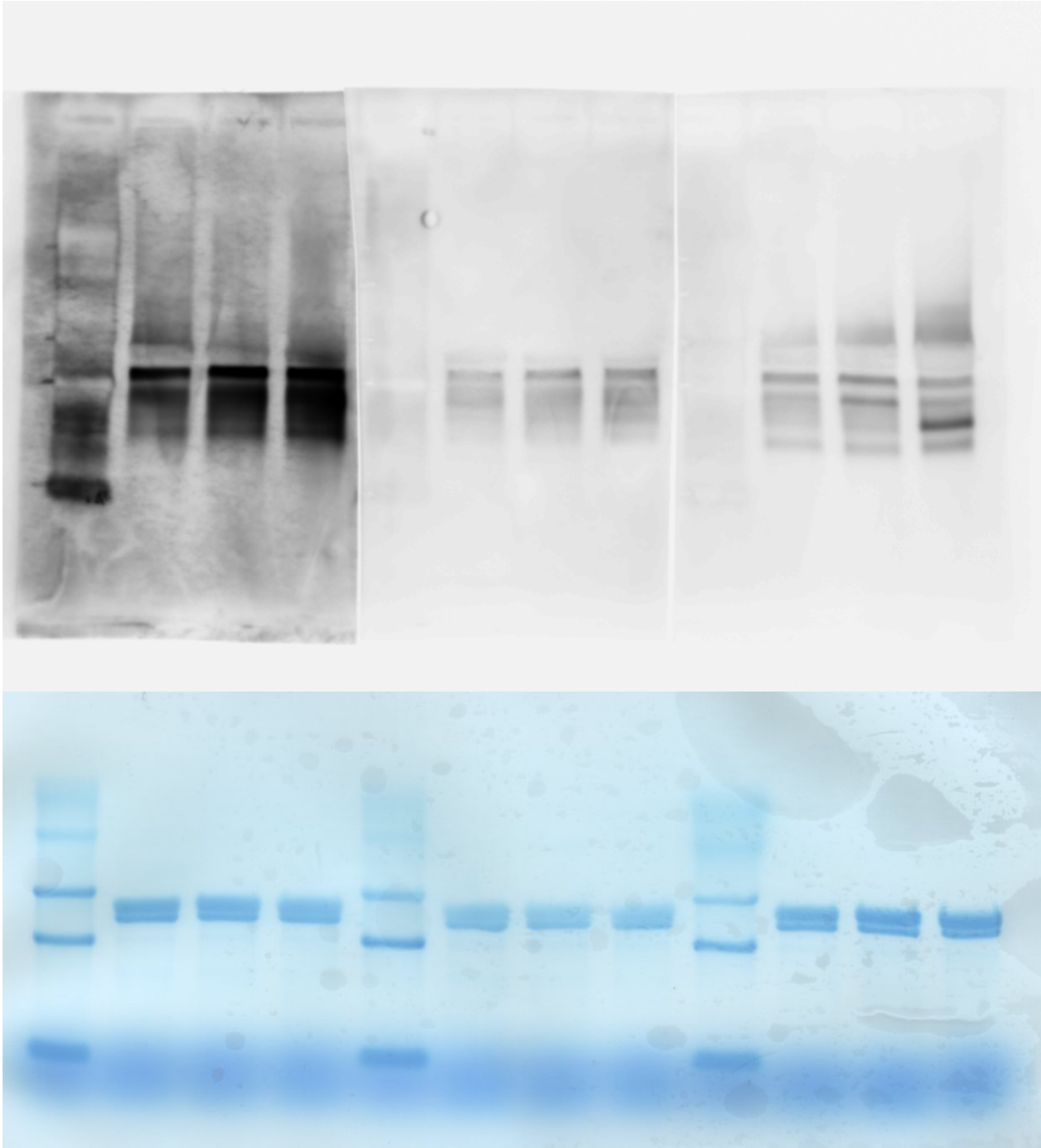

Supplementary Methods

Fly stocks

The following *Drosophila melanogaster* stocks were used in this study: *tsh-LacZ* (BL#11370), *esg-lacZ* (BL#10359) *Aph4-LacZ* (BL#12285), *esg-Gal4, UAS-GFP; tubulin-Gal80*^{ts}*/ SM6-TM6B*[1] (B. Edgar), *y, w, hsFLP, tubulin-Gal80 FR19A; UAS-mcd8::GFP/Cyo; tubulin-Gal4/TM6B,Tb* (N. Tapon*), esg-Gal4,UAS-mcd8::GFP/Cyo; UAS-H2B::RFP, tubulin-Gal80*^{ts}*/TM2*[2] (M. Dominguez)*, col-Gal4, UAS-mcd8::GFP/Cyo* and *dome-MESO-Gal4* (M. Crozatier), *GMR-Gal4/Cyo* (BL#9146), *tal-Gal4/TM3, Sb* (J.P. Couso), *c507-Gal4* (referred to as *Aph4-Gal4,* J. Dow)*, c724-Gal4* (J. Dow)*, G-TRACE (BL# 28280), svb*^{E}*-GFP, svb*^{E10}*-lacZ, svb*^{E3N}*-lacZ, svb*^{E6}*-lacZ* (D. Stern), *svb*^{E3N}*-GFP* (this publication), *svb*^{R9}*, FRT19A/FMO , svb*^{PL107}*, FRT19A/FMO*[3]*, ubr3*^{B}*,FRT19A/FMO*[4] (H. Bellen), *y, w, FRT82B, wts*^{X1}*/TM3, Sb* (N. Tapon), *w, FRT82B, pri*$^{S18.1}$/TM6B (J.P. Couso), *Diap1-lacZ* (BL#12093), *UAS-Cherry-RNAi* (BL# 35785), *UAS-svb-RNAi* (VDRC # 41584, TRIP GL00335)*, UAS-ubr3-RNAi* (VRDC #22901,#106993, #45166)*, UAS-yki-RNAi* (VDRC #KK104523, TRIP #HMS00041)*, UAS-tsh-RNAi (BL# 28022), UAS-pri-RNAi* (J.P. Couso)*, UAS-OvoA* [3], *UAS-OvoB*[3], *UAS-OvoA::GFP*, *UAS-OvoB::GFP* [5], *UAS-Svb-PPxY::GFP* (this publication), *UAS-EcRDN* (BL#9449), *UAS-mir8* (S.M. Cohen), *UAS-p35* (B. Monier), *UAS-hpo/CyO* (N. Tapon), *UAS-yki/TM3, Sb* (D.J. Pan), *UAS-yki::GFP* (BL#28815), *UAS-DIAP1* (N. Tapon), *UAS-pri/CyO* (J.P. Couso), *UAS-rpr* (BL# 5823).

Full genotypes

Figure 1a: *y, w/w; esg-Gal4, UAS-GFP/ tsh-LacZ*

Figure 1b: *y, w/w; esg-Gal4, UAS-GFP*

Figure 1c: *y, w/w; esg-LacZ/+; svb*^{E3N}*-GFP/+*

Figure 2a-c *control*: *y, w/w; esg-Gal4, UAS-GFP/+; tubulinGal80*^{ts}*/+*

Figure 2a-c *svb-RNAi:* *y, w/w; esg-Gal4, UAS-GFP/+; tubulin-Gal80*^{ts}*/ UAS-svb-RNAi*

Figure 2d *control*: *y, w, hsFLP, tubulin-Gal80, FR19A/ FRT19A; UAS-mCD8::GFP/+; tubulin-Gal4/ ry*^{506}

Figure 2d *svb*^{R9}: *y, w, hsFLP, tubulin-Gal80, FR19A/ y, w, svb*^{R9}*, FRT19A; UAS-mCD8::GFP/+; tubulin-Gal4/+*

Figure 3b: *tal-Gal4/ UAS-HB2::RFP*

Figure 3c *control*: *y, w/w; esg-Gal4, UAS-GFP/+; tubulin-Gal80*^{ts}*/ +*

Figure 3c *svb-RNAi*: *y, w/w; esg-Gal4, UAS-GFP/+; tubulin-Gal80*^{ts}*/ UAS-svb-RNAi*

Figure 3c *ovoA*: *y, w/w; esg-Gal4, UAS-GFP/+; tubulin-Gal80*^{ts}*/ UAS-ovoA*

Figure 3c *ovoB*: *y, w/w; esg-Gal4, UAS-GFP/+; tubulin-Gal80*^{ts}*/ UAS-ovoB*

Figure 3c *pri-RNAi*: *y, w/w; esg-Gal4, UAS-GFP/+; tubulin-Gal80*^{ts}*/ UAS-pri-RNAi*

Figure 3c *EcRDN*: *y, w/w; esg-Gal4, UAS-GFP/+; tubulin-Gal80*^{ts}*/ UAS-EcRDN*^{B2w650A}

Figure 3c *ubr3-RNAi*: *y, w/w; esg-Gal4, UAS-GFP/ UAS-ubr3-RNAi; tubulin-Gal80*^{ts}*/ +*

Figure 3c *ubr3-RNAi* + *ovoB*: *y, w/w; esg-Gal4, UAS-GFP/ ubr3-RNAi; tubulin-Gal80*^{ts}*/ UAS-ovoB/+*

Figure 3d *control*: *y, w, hsFLP, tubulin-Gal80, FR19A/ FRT19A; UAS-mCD8::GFP/+; tubulin-Gal4/ ry*^{506}

Figure 3d *ubr3*^{null}: *y, w, hsFLP, tubulin-Gal80, FR19A/ y,w, ubr3*^{B,} *FRT19A; UAS-mCD8::GFP/+; tubulin-Gal4/+*

Figure 4a *control*: *y, w/w; esg-Gal4, UAS-mCD8::GFP/+; UAS-H2B::RFP, tubulin-Gal80*^{ts}*/+*

Figure 4a *mir8*: *y, w/w, UAS-mir8; esg-Gal4, UAS-mCD8::GFP/+; UAS-H2B::RFP, tubulin-Gal80*^{ts}*/+*

Figure 4a *svb-RNAi*: *y, w/w; esg-Gal4, UAS-mCD8::GFP/+; UAS-H2B::RFP, tubulin-Gal80*^{ts}*/ UAS-svb-RNAi*

Figure 4b *control*: *y, w/w; esg-Gal4, UAS-GFP/+; tubulin-Gal80*^{ts}*/ +*

Figure 4b *p35*: *y, w/; esg-Gal4, UAS-GFP/ UAS-p35; tubulin-Gal80^{ts}/+*

Figure 4b *svb-RNAi*: *y, w/w; esg-Gal4, UAS-GFP/+; tubulin-Gal80^{ts}/ UAS-svb-RNAi*

Figure 4b *p35+ svb-RNAi*: *y, w/; esg-Gal4, UAS-GFP/ UAS-p35; tubulin-Gal80^{ts}/ UAS-svb-RNAi*

Figure 4c *control*: *y, w/w; esg-Gal4, UAS-GFP/+; tubulin-Gal80^{ts}/ +*

Figure 4c *>>Rpr*: *y, w/w UAS-rpr; esg-Gal4, UAS-GFP/+; tubulin-Gal80^{ts}/ +*

Figure 4c *svb-RNAi*: *y, w/w; esg-Gal4, UAS-GFP/+; tubulin-Gal80^{ts}/ UAS-svb-RNAi*

Figure 5 *control*: *y, w/w; esg-Gal4, UAS-GFP/+; tubulin-Gal80^{ts}/ +*

Figure 5 *hpo*: *y, w/w; esg-Gal4, UAS-GFP/ UAS-hpo; tubulin-Gal80^{ts}/+*

Figure 5 *yki*: *y, w/w; esg-Gal4, UAS-GFP/+; tubulin-Gal80^{ts}/ UAS-yki*

Figure 5 *DIAP1*: *y, w/w; esg-Gal4, UAS-GFP/ +; tubulin-Gal80^{ts}/ UAS-DIAP1*

Figure 5 *svb-RNAi*: *y, w/w; esg-Gal4, UAS-GFP/ +; tubulin-Gal80^{ts}/ UAS-svb-RNAi*

Figure 5 *hpo+ ovoB*: *y, w/w; esg-Gal4, UAS-GFP/ UAS-hpo; tubulin-Gal80^{ts}/ UAS-ovoB*

Figure 5 *yki+ svb-RNAi*: *y, w/w; esg-Gal4, UAS-GFP/ +; tubulin-Gal80^{ts}/ UAS-ovoB, UAS-yki.*

Figure 5 *yki+ ovoA*: *y, w/w; esg-Gal4, UAS-GFP/ +; tubulin-Gal80^{ts}/ UAS-yki, UAS-ovoA.*

Figure 5 *DIAP1+ svb-RNAi*: *y, w/w; esg-Gal4, UAS-GFP/ +; tubulin-Gal80^{ts}/ UAS-DIAP1, UAS-svb-RNAi*

Figure 6b *control*: *y, w/w; esg-Gal4, tubulin-Gal80^{ts}; DIAP1-LacZ/+*

Figure 6b *yki*: *y, w/w; esg-Gal4, tubulin-Gal80^{ts}; DIAP1-LacZ/ UAS-yki*

Figure 6b *ovoA*: *y, w/w; esg-Gal4, tubulin-Gal80^{ts}; DIAP1-LacZ/ UAS-ovoA*

Figure 6b *yki + ovoA*: *y, w/w; esg-Gal4, tubulin-Gal80^{ts}; DIAP1-LacZ/ UAS-yki, UAS-ovoA*

Figure 6b *control*: *y, w/w; esg-Gal4, tubulin-Gal80^{ts}/ +; DIAP14.3-GFP/+*

Figure 6b *yki*: *y, w/w; esg-Gal4, tubulin-Gal80^{ts}/+; DIAP14.3-GFP / UAS-yki*

Figure 6b *ovoA*: *y, w/w; esg-Gal4, tubulin-Gal80^{ts}/ +; DIAP14.3-GFP / UAS-ovoA*

Figure 6b *yki + ovoA*: *y, w/w; esg-Gal4, tubulin-Gal80^{ts}/+; DIAP14.3-GFP / UAS-yki, UAS-ovoA*

Figure 7b *ovoB::GFP*: *y, w/w; esg-Gal4, tubulin-Gal80^{ts}/ UAS-ovoB::GFP*

Figure 7b *ovoA::GFP*: *y, w/w; esg-Gal4, tubulin-Gal80^{ts}/ UAS-ovoA::GFP*

Figure 7b *svbPPxY::GFP*: *y, w/w; esg-Gal4, tubulin-Gal80^{ts}/ UAS-svb-PPxY-6C::GFP*

Figure 7b *svbPPxY::GFP*: *y, w/w; esg-Gal4, tubulin-Gal80^{ts}/+; UAS-svb-PPxY-9C::GFP/+*

Figure 8a *control*: *y,w/w; col-Gal4, UAS-mCD8::GFP/+*

Figure 8a *yki*: *y,w/w; col-Gal4, UAS-mCD8::GFP/+; UAS-yki /+*

Figure 8a *ovoA*: *y,w/w; col-Gal4, UAS-mCD8::GFP/+; UAS-ovoA/+*

Figure 8a *yki + ovoA*: *y,w/w; col-Gal4, UAS-mCD8::GFP/+; UAS-yki, UAS-ovoA/+*

Figure 8a *svbPPxY*: *y,w/w; col-Gal4, UAS-mCD8::GFP/+; UAS-svb-PPxY::GFP /+*

Figure 8b *ctrl*: *y,w/w; FRT82B/+*

Figure 8b *pri/+*: *y,w/w; FRT82B,* $pri^{S18.1}$*/+*

Figure 8b *wts/+*: *y,w/y,w; FRT82B,* wts^{X1} */+*

Figure 8b *pri/+, wts/+*: *y,w/w; FRT82B,* $pri^{S18.1}$*,* wts^{X1} */+*

Figure Sup1b: *y, w/w; esg-LacZ/*svb^{E10}*-GFP*

Figure Sup1c: *y, w/w; esg-LacZ/+*

Figure Sup1c*:* *w;* svb^{E3N}*-LacZ/+*

Figure Sup1c: *w;* svb^{E6}*-LacZ/+*

Figure Sup2a *control*: *y, w/w; esg-Gal4, UAS-GFP/+; tubulin-*$Gal80^{ts}$*/+*

Figure Sup2a *svb-RNAi*: *y, w/w; esg-Gal4, UAS-GFP/+; tubulin-*$Gal80^{ts}$*/ UAS-svb-RNAi (VDRC#41584)*

Figure Sup2b *svb-RNAi*: *y, w/w; esg-Gal4, UAS-GFP/+; tubulin-*$Gal80^{ts}$*/ UAS-svb-RNAi (TRIP#GL00335)*

Figure Sup2c: *y, w/w; esg-Gal4, UAS-GFP/+; tubulin-*$Gal80^{ts}$*/+*

Figure Sup2d *control*: *y, w/w; esg-Gal4, UAS-GFP/+; tubulin-*$Gal80^{ts}$*/+*

Figure Sup2d *svb-RNAi*: *y, w/w; esg-Gal4, UAS-GFP/+; tubulin-*$Gal80^{ts}$*/ UAS-svb-RNAi*

Figure Sup2e *control*: *y,w/w; dome-MESO-Gal4, UAS-mCherry/+*

Figure Sup2e *svb-RNAi*: *y,w/w; dome-MESO-Gal4, UAS-mCherry/+; UAS-svb-RNAi/+*

Figure Sup2f *control*: *y,w, hsFLP, tubulin-Gal80,FR19A/ FRT19A; UAS-mCD8::GFP/+; tubulin-Gal4/* ry^{506}

Figure Sup2f svb^{PL107}**:** *y,w, hsFLP, tubulin-Gal80, FR19A/ y,w,* $svb^{PL107,}$ *FRT19A; UAS-mCD8::GFP/+; tubulin-Gal4/+*

Figure Sup2g *control*: *y,w, hsFLP, tubulin-Gal80,FR19A/ FRT19A; UAS-mCD8::GFP/+; tubulin-Gal4/* ry^{506}

Figure Sup2g svb^{R9}**:** *y,w, hsFLP, tubulin-Gal80 FR19A/ y,w,* $svb^{R9,}$ *FRT19A; UAS-mCD8::GFP/+; tubulin-Gal4/+*

Figure Sup3b *esg-LacZ*: *y,w/w; esg-LacZ/+*

Figure Sup3b *priA-LacZ*: *y,w; priA-LacZ/+*

Figure Sup3b *priJ-LacZ*: *y,w/+; priJ-LacZ/+*

Figure Sup3c *control*: *y,w/w; esg-Gal4, UAS-GFP/+; tubulin-Gal80^{ts}/ +*

Figure Sup3c *svb-RNAi*: *y,w/w; esg-Gal4, UAS-GFP/+; tubulin-Gal80^{ts}/ UAS-svb-RNAi*

Figure Sup3c *pri-RNAi*: *y,w/w; esg-Gal4, UAS-GFP/+; tubulin-Gal80^{ts}/ UAS-pri-RNAi*

Figure Sup3d *control*: *y,w/w; esg-Gal4, UAS-GFP/+; tubulin-Gal80^{ts}/+*

Figure Sup3d *cherry-IR*: *y,w/yv; esg-Gal4, UAS-GFP/+; tubulin-Gal80^{ts}/ UAS-Cherry-RNAi*

Figure Sup3d *yki-IR HMS*: *y,w/y,w; esg-Gal4, UAS-GFP/ +; tubulin-Gal80^{ts}/ UAS-yki-RNAi^{HMS00041}*

Figure Sup3d *ubr3-IR 106993*: *y,w/w; esg-Gal4, UAS-GFP/ UAS-ubr3-RNAi$^{VDRC\#106993}$; tubulin-Gal80^{ts}/ +*

Figure Sup3d *ubr3-IR 45166*: *y,w/w; esg-Gal4, UAS-GFP/ UAS-ubr3-RNAi $^{VDRC\#45166}$; tubulin-Gal80^{ts}/ +*

Figure Sup4a: *y,w/y,w; esg-Gal4, UAS-GFP/*

Figure Sup4b *ctrl*: *w; c724-Gal4/+*

Figure Sup4b *Tsh RNAi*: *w/w; c724-Gal4/+; UAS-Tsh-RNAi$^{JFO\ 2856}$*

Figure Sup4c *dome-Gal4*: *w; UAS-Red-Stinger, UAS-Flp, ubi63(FRT-Stop)Stinger/ dome-MESO-Gal4*

Figure Sup4c *esg-Gal4*: *w; UAS-RedStinger, UAS-Flp, ubi63(FRT-Stop)Stinger/ esg-Gal4*

Figure Sup4c *Aph4-Gal4*: *w; UAS-RedStinger, UAS-Flp, ubi63(FRT-Stop)Stinger/+ ; c507-Gal4*

Figure Sup4d *esg-Gal4*: *w; UAS-RedStinger, UAS-Flp, ubi63(FRT-Stop)Stinger/ esg-Gal4*

Figure Sup5a *control*: *y, w/w; esg-Gal4, UAS-mCD8::GFP/+; UAS-H2B::RFP, tubulin-Gal80^{ts}/ +*

Figure Sup5a *>mir8*: *y, w/w, UAS-mir8; esg-Gal4, UAS-mCD8::GFP/+; UAS-H2B::RFP, tubulin-Gal80^{ts}/ +*

Figure Sup5a *svb-RNAi*: *y, w/w; esg-Gal4, UAS-mCD8::GFP/+; UAS-H2B::RFP, tubulin-Gal80^{ts}/ UAS-svb-RNAi*

Figure Sup5b *control*: *y,w/w; esg-Gal4, UAS-mCD8::GFP/+; UAS-H2B::RFP, tubulin-Gal80^{ts}/ Aph4-LacZ^{07028}, ry*

Figure Sup5b >*mir-8*: *y,w/w, UAS-mir8; esg-Gal4, UAS-mCD8::GFP/+; UAS-H2B::RFP, tubulin-Gal80^{ts}/Aph4-LacZ^{07028}, ry*

Figure Sup6a *control*: *y,w/w; esg-Gal4, UAS-GFP/+; tubulin-Gal80^{ts}/+*

Figure Sup6a *ovoB*: *y,w/w; esg-Gal4, UAS-GFP/ +; tubulin-Gal80^{ts}/UAS-ovoB*

Figure Sup6a *yki-RNAi*: *y,w/w; esg-Gal4, UAS-GFP/ / UAS-yki-RNAi; tubulin-Gal80^{ts}/+*

Figure Sup6a *yki-RNAi* + *ovoB*: *y,w/w; esg-Gal4, UAS-GFP/ UAS-yki-RNAi; tubulin-Gal80^{ts}/ UAS-ovoB*

Figure Sup6b *control*: *y,w/w; esg-Gal4, tubulin-Gal80^{ts}/+; DIAP14.3-GFP/+*

Figure Sup6b *ovoB*: *y,w/w; esg-Gal4, tubulin-Gal80^{ts}/+; DIAP14.3-GFP/ UAS-ovoB*

Figure Sup6c *control*: *y,w/w; esg-Gal4, tubulin-Gal80^{ts}/ UAS-Yki::GFP*

Figure Sup6c *svb RNAi*: *y,w/w; esg-Gal4, tubulin-Gal80^{ts}/ UAS-Yki::GFP; UAS-svb-RNAi /+*

Figure Sup6c *ovoA*: *y,w/w; esg-Gal4, tubulin-Gal80^{ts}/ UAS-Yki::GFP; UAS-ovoA /+*

Figure Sup6c *ovoB*: *y,w/w; esg-Gal4, tubulin-Gal80^{ts}/ UAS-Yki::GFP; UAS-ovoB /+*

Figure Sup8a *ctrl*: *y,w/w; ptc-Gal4/+*

Figure Sup8a *ovoA*: *y,w/w; ptc-Gal4/+; UAS-ovoA::GFP*

Figure Sup8a *ovoB*: *y,w/w; ptc-Gal4/+; UAS-ovoB::GFP*

Figure Sup8a *ctrl*: *y,w/w; ptc-Gal4/+; UAS-svb-PPXY::GFP*

Figure Sup8b *ctrl*: *sd^{ETx4}/Y; FRT82B /+*

Figure Sup8b *wts/+*: *sd^{ETx4}/Y; FRT82B, wts^{X1} /+*

Figure Sup8b *pri/+, wts/+*: *sd^{ETx4}/Y; FRT82B, pri$^{S18.1}$, wts^{X1} /+*

Figure Sup9a *ctrl*: *y,w/w; GMR-Gal4/+*

Figure Sup9a *pri*: *y,w/w; GMR-Gal4/UAS-pri*

Figure Sup9a *yki*: *y,w/w; GMR-Gal4/+; UAS-yki*

Figure Sup9a *pri+ yki*: *y,w/w; GMR-Gal4/UAS-pri; UAS-yki/+*

Figure Sup9b *wt*: *Oregon-R*

Figure Sup9b *GMR*: *y,w/w; GMR-Gal4/+*

Figure Sup9b GMR>*pri*: *y,w/w; GMR-Gal4/UAS-pri*

ChIP-seq analysis

Raw data (.fq files) of ChIPseq obtained for Yki [6] (GSM945893 GSM945894) and Svb[7] (GSM1184656, GSM1184657, GSM1184658) were processed in parallel, using the same pipeline. Briefly, reads were aligned to the *Drosophila* genome release Dm6 with BWA[8]. SAM files were converted into BAM files using SAMtools[9-11] and further processed for peak calling. The quality of sequencing was checked using FastQC, and we used SAMTOOLS *flagstat* to calculate the percentage of mapped reads to estimate the quality of alignment from the percentage of mapped reads (as indicated below).

Number of reads	FastQC	BAM files	Mapping
Svb_input	18,523,111	13,322,332	71,92 %
Svb_Rep#1	16,956,734	12,715,257	74,99 %
Svb_Rep#2	61,296,665	46,185,543	75,35 %
Yki_input	41,901,421	25,549,865	60,98 %
Yki_#1	29,985,092	22,041,362	73,51 %

Peaks were called using MACS2[12], using a p value of 0.1. The package ChIPpeakAnno[13] (available under RBioconductor) was used to identify co-binding regions of Svb and Yki, and to perform permutation-statistical tests[13]. An UCSC genome browser is available at:

http://genome-euro.ucsc.edu/cgi-bin/hgTracks?hgS_doOtherUser=submit&hgS_otherUserName=amanchenoferris&hgS_otherUserSessionName=bohere_etal

DNA constructs and mutagenesis

The DNA region encoding point mutations to inactivate the two PPxY motifs of Svb (>PPxA) has been optimized to be chemically synthetized (Integrated DNA Technologies). Resulting DNA was amplified by PCR and cloned into the pAc-Svb::GFP construct[4], linearized with PmlI and HindIII restriction sites, using the In-Fusion HD Cloning kit (Clontech laboratories, Inc) to obtain the pAc-Svb-PPxY::GFP vector. The EcoRI fragment with both PPxA mutated sites from pAc-Svb-PPxY::GFP has been inserted into the pUASp-Svb::GFP[5] linearized with EcoRI. All construct sequences have been verified by sequencing.

NORTHERN blotting

Using adult total RNAs as a starting material, DNA fragments containing coding sequence of *yki*, *CycE* and *DIAP1* were reverse transcribed and PCR amplified. An SP6 promoter sequence (CAAGC TATTT AGGTG ACACT ATAG) was attached to each reverse primer for *in vitro* transcription. DIG-labelled probes were prepared with SP6 RNA polymerase, according to the supplier's manual (Roche).

Oligonucleotides

**Oligonucleotides PPxY mutagenesis**		
5-TCAGCTCAGCATGCCACGCGTG-3	Integrated DNA Technologies	3'Ex2-Fw
5-CGGCAAGCCCAAGCTTTGGATGAGTGGC-3	Integrated DNA Technologies	3'Ex2-Rev
**Oligonucleotides Northern blotting**		
5-CTGCCCAACTCCTTCTTCAC-3	Integrated DNA Technologies	Yki Forward
5-AACTGAATGGGGCTGATGAC-3	Integrated DNA Technologies	Yki Reverse
5-GATGACGTTGAGGAGGAGGA-3	Integrated DNA Technologies	CycE Forward
5-TGCGTCTTCTGCACCTTATG-3	Integrated DNA Technologies	CycE Reverse
5-CCGAGGAACCTGAAACAGAA-3	Integrated DNA Technologies	DIAP1Forward
5-GCACAACTTTTCCTCGGGTA-3	Integrated DNA Technologies	DIAP1Reverse

Supplementary References

1. Jiang, H. *et al.* Cytokine/Jak/Stat Signaling Mediates Regeneration and Homeostasis in the Drosophila Midgut. *Cell* **137**, 1343-1355 (2009).
2. Antonello, Z.A., Reiff, T., Esther, B.-I. & Dominguez, M. Robust intestinal homeostasis relies on cellular plasticity in enteroblasts mediated by *miR-8-Escargot* switch. *EMBO J.* **34**, 2025-2041 (2015).
3. Delon, I., Chanut-Delalande, H. & Payre, F. The Ovo/Shavenbaby transcription factor specifies actin remodelling during epidermal differentiation in Drosophila. *Mech Dev* **120**, 747-758 (2003).
4. Zanet, J. *et al.* Pri sORF peptides induce selective proteasome-mediated protein processing. *Science* **349**, 1356-1358 (2015).
5. Kondo, T. *et al.* Small peptides switch the transcriptional activity of Shavenbaby during Drosophila embryogenesis. *Science* **329**, 336-339 (2010).
6. Oh, H. *et al.* Genome-wide association of Yorkie with chromatin and chromatin-remodeling complexes. *Cell Rep* **3**, 309-318 (2013).
7. Menoret, D. *et al.* Genome-wide analyses of Shavenbaby target genes reveals distinct features of enhancer organization. *Genome Biol* **14**, R86 (2013).
8. Li, H. & Durbin, R. Fast and accurate short read alignment with Burrows-Wheeler transform. *Bioinformatics* **25**, 1754-1760 (2009).
9. Etherington, G.J., Ramirez-Gonzalez, R.H. & MacLean, D. bio-samtools 2: a package for analysis and visualization of sequence and alignment data with SAMtools in Ruby. *Bioinformatics* **31**, 2565-2567 (2015).
10. Kaisers, W., Schaal, H. & Schwender, H. rbamtools: an R interface to samtools enabling fast accumulative tabulation of splicing events over multiple RNA-seq samples. *Bioinformatics* **31**, 1663-1664 (2015).
11. Li, H. *et al.* The Sequence Alignment/Map format and SAMtools. *Bioinformatics* **25**, 2078-2079 (2009).
12. Zhang, Y. *et al.* Model-based analysis of ChIP-Seq (MACS). *Genome Biol* **9**, R137 (2008).
13. Zhu, L.J. *et al.* ChIPpeakAnno: a Bioconductor package to annotate ChIP-seq and ChIP-chip data. *BMC Bioinformatics* **11**, 237 (2010).
