# Supplementary material for: Shavenbaby and Yorkie mediate Hippo signaling to protect adult stem cells from apoptosis
Source: Nat Commun. 2018 Nov 30;9:5123. doi: 10.1038/s41467-018-07569-0 (PMC6269459; doi:10.1038/s41467-018-07569-0)
Supplement: Supplementary file 3 — Description of Additional Supplementary files [file 41467_2018_7569_MOESM3_ESM.pdf]

Supplementary Data 1: ChIP-seq peaks for Svb and Yki
