# Supplementary material for: Shavenbaby and Yorkie mediate Hippo signaling to protect adult stem cells from apoptosis
Source: Nat Commun. 2018 Nov 30;9:5123. doi: 10.1038/s41467-018-07569-0 (PMC6269459; doi:10.1038/s41467-018-07569-0)
Supplement: Supplementary file 5 — Reporting Summary [file 41467_2018_7569_MOESM5_ESM.pdf]

nature research

Corresponding author(s): C. POLESELLO & F. PAYRE

Reporting Summary

Nature Research wishes to improve the reproducibility of the work that we publish. This form provides structure for consistency and transparency in reporting. For further information on Nature Research policies, see Authors & Referees and the Editorial Policy Checklist.

Statistical parameters

When statistical analyses are reported, confirm that the following items are present in the relevant location (e.g. figure legend, table legend, main text, or Methods section).

n/a	Confirmed	
☐	☒	The exact sample size (n) for each experimental group/condition, given as a discrete number and unit of measurement
☐	☒	An indication of whether measurements were taken from distinct samples or whether the same sample was measured repeatedly
☐	☒	The statistical test(s) used AND whether they are one- or two-sided<br>*Only common tests should be described solely by name; describe more complex techniques in the Methods section.*
☒	☐	A description of all covariates tested
☒	☐	A description of any assumptions or corrections, such as tests of normality and adjustment for multiple comparisons
☐	☒	A full description of the statistics including central tendency (e.g. means) or other basic estimates (e.g. regression coefficient) AND variation (e.g. standard deviation) or associated estimates of uncertainty (e.g. confidence intervals)
☐	☒	For null hypothesis testing, the test statistic (e.g. F, t, r) with confidence intervals, effect sizes, degrees of freedom and P value noted<br>*Give P values as exact values whenever suitable.*
☒	☐	For Bayesian analysis, information on the choice of priors and Markov chain Monte Carlo settings
☒	☐	For hierarchical and complex designs, identification of the appropriate level for tests and full reporting of outcomes
☒	☐	Estimates of effect sizes (e.g. Cohen's d, Pearson's r), indicating how they were calculated
☐	☒	Clearly defined error bars<br>*State explicitly what error bars represent (e.g. SD, SE, CI)*

Our web collection on statistics for biologists may be useful.

Software and code

Policy information about availability of computer code

Data collection	N/A
Data analysis	BWA, SAMTOOLS, MACS2, ChIPpeakAnno, RBioconductor, Prism 5, Imaris 8.0, Photoshop CS5, Image J

For manuscripts utilizing custom algorithms or software that are central to the research but not yet described in published literature, software must be made available to editors/reviewers upon request. We strongly encourage code deposition in a community repository (e.g. GitHub). See the Nature Research guidelines for submitting code & software for further information.

Data

Policy information about availability of data

All manuscripts must include a data availability statement. This statement should provide the following information, where applicable:

- Accession codes, unique identifiers, or web links for publicly available datasets
- A list of figures that have associated raw data
- A description of any restrictions on data availability

The datasets analyzed during the current study are available in the EMBL-EBI repository,
The data that support the findings of this study are given in Supplementary Table 1.
Detailed scripts and computer code used for ChIPseq analysis are available from the corresponding author on request.

Field-specific reporting

Please select the best fit for your research. If you are not sure, read the appropriate sections before making your selection.

☒ Life sciences ☐ Behavioural & social sciences ☐ Ecological, evolutionary & environmental sciences

For a reference copy of the document with all sections, see nature.com/authors/policies/ReportingSummary-flat.pdf

Life sciences study design

All studies must disclose on these points even when the disclosure is negative.

Sample size	The typical sample size is >20, the number of samples is given in each figure and supplementary figure.
Data exclusions	No data were excluded.
Replication	All experiments have been replicated using 2 or 3 independant experiments.
Randomization	N/A
Blinding	N/A

Reporting for specific materials, systems and methods

Materials & experimental systems

n/a	Involved in the study
☐	☐ Unique biological materials
☐	☒ Antibodies
☐	☒ Eukaryotic cell lines
☐	☐ Palaeontology
☐	☒ Animals and other organisms
☐	☐ Human research participants

Methods

n/a	Involved in the study
☐	☒ ChIP-seq
☐	☐ Flow cytometry
☐	☐ MRI-based neuroimaging

Unique biological materials

Policy information about availability of materials

Obtaining unique materials	N/A

Antibodies

Antibodies used

Rabbit anti ß-Galactosidase - MP Biomedicals (Cappel) - Cat#08559761
Mouse anti Cut - Developmental Studies Hybridoma Bank - Cat#2B10
Mouse anti Dlg - Developmental Studies Hybridoma Bank Cat#4F3 anti-discs large
Mouse anti-Arm - Developmental Studies Hybridoma Bank - Cat# N2 7A1
Mouse anti-Hnt - Developmental Studies Hybridoma Bank - Cat# 1G9
Rabbit anti- GFP- TP401, Acris Antibodies
Rabbit ant Phosphorylated Histone H3 (Ser 10) - Cell Signalling Upstate Cat#TAKMA312B
Rabbit anti GFP - Acris Antibodies Cat#TP401
GFP-binding protein (GFP-trap) Chromotek Cat#gt250
Mouse anti HA - Covance - Cat#BLE901513
Rabbit anti-Yki - kind gift of K. Irvine
Alexa Fluor Rabbit 488 - ThermoFischer Scientific - Cat#Z25302
Alexa Fluor Mouse 555 - ThermoFischer Scientific - Cat#Z25205
Anti IgG-HRP - Jackson Laboratory - Cat#515-035-003

Validation

Rabbit anti ß-Galactosidase - https://www.mpbio.com/product.php?pid=0855976&country=223

Mouse anti Cut - http://dshb.biology.uiowa.edu/2B10
Mouse anti Dlg - http://dshb.biology.uiowa.edu/4F3-anti-discs-large
Mouse anti-Arm -http://dshb.biology.uiowa.edu/N2-7A1-ARMADILLO
Mouse anti-Hnt - http://dshb.biology.uiowa.edu/hindsight-protein
Rabbit anti- GFP- https://www.acris-anticorps.fr/antibodies/primary-antibodies/gfp-tp401.htm
GFP-binding protein (GFP-trap) https://www.chromotek.com/products/nano-traps/gfp-trap/gfp-trapr-a/
Mouse anti HA - https://www.biolegend.com/en-us/products/purified-anti-ha-11-epitope-tag-antibody-11374
Rabbit anti-Yki- Oh and Irvine- Development 2008- doi: 10.1242/dev.015255

Eukaryotic cell lines

Policy information about cell lines

Cell line source(s)	Drosophila S2 cell line (DGRC) and 1B and FS lines-<br>Zanet, J. et al. Pri sORF peptides induce selective proteasome-mediated protein processing. Science 349, 1356-1358 (2015).
Authentication	None of the cell lines used have been authentificated.
Mycoplasma contamination	the cell lines have not been tested for mycoplasma
Commonly misidentified lines<br>(See ICLAC register)	N/A

Palaeontology

Specimen provenance	N/A
Specimen deposition	N/A
Dating methods	N/A

☐ Tick this box to confirm that the raw and calibrated dates are available in the paper or in Supplementary Information.

Animals and other organisms

Policy information about studies involving animals; ARRIVE guidelines recommended for reporting animal research

Laboratory animals

tsh-LacZ (BL#11370),
esg-lacZ (BL#10359),
Aph4-LacZ (BL#12285),
esg-Gal4, UAS-GFP; tubulin-Gal80ts/ SM6-TM6B1 (B. Edgar),
y, w, hsFLP, tubulin-Gal80 FR19A; UAS-mcd8::GFP/Cyo; tubulin-Gal4/TM6B,Tb (N. Tapon),
esg-Gal4,UAS-mcd8::GFP/Cyo; UAS-H2B::RFP, tubulin-Gal80ts/TM22 (M. Dominguez),
col-Gal4, UAS-mcd8::GFP/Cyo and dome-MESO-Gal4 (M. Crozatier),
GMR-Gal4/Cyo (BL#9146),
tal-Gal4/TM3, Sb (J.P. Couso),
c507-Gal4 (referred to as Aph4-Gal4, J. Dow),
c724-Gal4 (J. Dow),
G-TRACE (BL# 28280),
svbE-GFP, svbE10-lacZ, svbE3N-lacZ, svbE6-lacZ (D. Stern),
svbR9, FRT19A/FMO , svbPL107, FRT19A/FMO (Delon et al., Mech Dev 120, 747-758 (2003).)
ubr3B,FRT19A/FMO4 (H. Bellen),
y, w, FRT82B, wtsX1/TM3, Sb (N. Tapon),
w, FRT82B, priS18.1/TM6B (J.P. Couso),
Diap1-lacZ (BL#12093),
UAS-Cherry-RNAi (BL# 35785), UAS-svb-RNAi (VDRC # 41584, TRIP GL00335), UAS-ubr3-RNAi (VRDC #22901,#106993, #45166),
UAS-yki-RNAi (VDRC #KK104523, TRIP #HMS00041), UAS-tsh-RNAi (BL# 28022), UAS-pri-RNAi (J.P. Couso),
UAS-OvoA , UAS-OvoB , UAS-OvoA::GFP, UAS-OvoB::GFP (Kondo et al., Science 329, 336-339 (2010).)
UAS-Svb-PPxY::GFP (this publication),
UAS-EcRDN (BL#9449),
UAS-mir8 (S.M. Cohen),
UAS-p35 (B. Monier),
UAS-hpo/CyO (N. Tapon),
UAS-yki/TM3, Sb (D.J. Pan),
UAS-yki::GFP (BL#28815),
UAS-DIAP1 (N. Tapon),
UAS-pri/CyO (J.P. Couso),
UAS-rpr (BL# 5823)

Wild animals: N/A

Field-collected samples | N/A

Human research participants

Policy information about studies involving human research participants

Population characteristics | N/A

Recruitment | N/A

ChIP-seq

Data deposition

☐ Confirm that both raw and final processed data have been deposited in a public database such as GEO.

☒ Confirm that you have deposited or provided access to graph files (e.g. BED files) for the called peaks.

Data access links
May remain private before publication.

https://www.ebi.ac.uk/biosamples/samples/SAMEA2439952
https://www.ebi.ac.uk/biosamples/samples/SAMN01041482
https://www.ebi.ac.uk/biosamples/samples/SAMN02231205
https://www.ebi.ac.uk/biosamples/samples/SAMN02231213
https://www.ebi.ac.uk/biosamples/samples/SAMN02231206
Supplementary Table 1 contains the called peaks for Yki and Svb

Files in database submission | N/A

Genome browser session (e.g. UCSC)

http://genome-euro.ucsc.edu/cgi-bin/hgTracks?
hgS_doOtherUser=submit&hgS_otherUserName=amanchenoferris&hgS_otherUserSessionName=bohere_etal

Methodology

Replicates | all ChIP-seq data have been published previoulsy

Sequencing depth | N/A

Antibodies | N/A

Peak calling parameters | Peaks were called using MACS212, using a p value of 0.1. The package ChIPpeakAnno13 (available under RBioconductor) was used to identify co-binding regions of Svb and Yki, and to perform permutation-statistical tests13.

Data quality | The quality of sequencing was checked using FastQC, and we used SAMTOOLS flagstat to calculate the percentage of mapped reads to estimate the quality of alignment from the percentage of mapped reads (see data in sup mat)

Software | Raw data (.fq files) of ChIPseq obtained for Yki 6 (GSM945893 GSM945894) and Svb7 (GSM1184656, GSM1184657, GSM1184658) were processed in parallel, using the same pipeline. Briefly, reads were aligned to the Drosophila genome release Dm6 with BWA8. SAM files were converted into BAM files using SAMtools9-11 and further processed for peak calling.

Flow Cytometry

Plots

Confirm that:

☐ The axis labels state the marker and fluorochrome used (e.g. CD4-FITC).

☐ The axis scales are clearly visible. Include numbers along axes only for bottom left plot of group (a 'group' is an analysis of identical markers).

☐ All plots are contour plots with outliers or pseudocolor plots.

☐ A numerical value for number of cells or percentage (with statistics) is provided.

Methodology

Sample preparation | N/A

Instrument | N/A

Software | N/A

Cell population abundance	N/A
Gating strategy	N/A

☐ Tick this box to confirm that a figure exemplifying the gating strategy is provided in the Supplementary Information.

Magnetic resonance imaging

Experimental design

Design type	N/A
Design specifications	N/A
Behavioral performance measures	N/A

Acquisition

Imaging type(s)	N/A
Field strength	N/A
Sequence & imaging parameters	N/A
Area of acquisition	N/A

Diffusion MRI ☐ Used ☐ Not used

Preprocessing

Preprocessing software	N/A
Normalization	N/A
Normalization template	N/A
Noise and artifact removal	N/A
Volume censoring	N/A

Statistical modeling & inference

Model type and settings	N/A
Effect(s) tested	N/A

Specify type of analysis: ☐ Whole brain ☐ ROI-based ☐ Both

Statistic type for inference (See Eklund et al. 2016)	N/A
Correction	N/A

Models & analysis

n/a	Involved in the study
☒	☐ Functional and/or effective connectivity
☒	☐ Graph analysis
☒	☐ Multivariate modeling or predictive analysis
